# Genetic Diversity and Molecular Evolution of a Violaxanthin De-epoxidase Gene in Maize

**DOI:** 10.3389/fgene.2016.00131

**Published:** 2016-07-26

**Authors:** Jing Xu, Zhigang Li, Haorui Yang, Xiaohong Yang, Cuixia Chen, Hui Li

**Affiliations:** ^1^State Key Laboratory of Crop Biology, Shandong Key Laboratory of Crop Biology, Shandong Agricultural UniversityTai’an, China; ^2^Key Laboratory of Crop Genomics and Genetic Improvement, National Maize Improvement Center of China, China Agricultural UniversityBeijing, China; ^3^School of Biological Science and Technology, University of JinanJinan, China

**Keywords:** violaxanthin de-epoxidase, nucleotide diversity, selection, domestication, carotenoid, maize

## Abstract

Violaxanthin de-epoxidase (VDE) has a critical role in the carotenoid biosynthesis pathway, which is involved in protecting the photosynthesis apparatus from damage caused by excessive light. Here, a *VDE* gene in maize, *ZmVDE1*, was cloned and shown to have functional domains in common with the gramineous VDE protein. Candidate gene association analysis indicated that no polymorphic sites in *ZmVDE1* were significant association with any of the examined carotenoid-related traits at *P* = 0.05 in an association panel containing 155 maize inbred lines. Nucleotide diversity analysis of *VDE1* in maize and teosinte indicated that its exon had less genetic variation, consistent with the conserved function of *VDE1* in plants. In addition, dramatically reduced nucleotide diversity, fewer haplotypes and a significantly negative parameter deviation for Tajima’s *D* test of *ZmVDE1* in maize and teosinte suggested that a potential selective force had acted across the *ZmVDE1* locus. We further identified a 4.2 Mb selective sweep with low recombination surrounding the *ZmVDE1* locus that resulted in severely reduced nucleotide diversity on chromosome 2. Collectively, natural selection and the conserved domains of *ZmVDE1* might show an important role in the xanthophyll cycle of the carotenoid biosynthesis pathway.

## Introduction

Crop domestication and breeding, which have been ongoing for > 10,000 years, represent evolutionary experiments that have radically altered wild species to meet human needs (Hufford et al., 2012). Archeological (Piperno et al., 2009) and genetic (van Heerwaarden et al., 2011) evidence indicates that maize (*Zea mays* ssp. *mays*) was domesticated roughly 10,000 years ago in southern Mexico from Balsas teosinte (*Zea mays* ssp. *parviglumis*). Maize domestication involved a radical phenotypic transformation, resulting in an unbranched plant with numerous exposed seed attached to a cob in 20 rows or more. The dramatic morphological changes from teosinte to maize likely involved alterations in only a few significant genes with large effects (Tian et al., 2009).

Recent quantitative trait loci (QTL) mapping identified five regions of the maize genome with large effects on basic morphology (Doebley et al., 1990; Doebley and Stec, 1991), two of which have now been studied in great detail. One locus, *teosinte branched1* (*tb1*), which was a major contributor to the increase in apical dominance during maize domestication, has been successfully cloned (Doebley et al., 1995, 1997; Wang et al., 1999). A transposable element inserted 60 kb upstream of *tb1* acts as an enhancer of gene expression and partially explains the increased apical dominance in maize compared with teosinte (Studer et al., 2011). A single genetic locus, *teosinte glume architecture1* (*tga1*), has been identified as a QTL controlling the formation of the hardened protective covering on teosinte kernels that has mostly been lost in maize (Dorweiler et al., 1993). A single amino acid change within *tga1*, which belongs to the squamosa promoter-binding protein-like transcription factor (SBP-domain) family of transcriptional regulators, is responsible for this radical difference (Wang et al., 2005). Molecular evolutionary analyses have indicated that this region was the target of selection during maize domestication (Wang et al., 2005). Subsequently, a few genes underwent a process of genetic modification to meet the needs of humans, including an increase in harvestable yield and better kernel quality (Tian et al., 2009). Four of the six genes involved in the starch biosynthesis pathway show evidence of selection (Whitt et al., 2002).

Carotenoids are natural plant pigments that are widely found in plants, although among cereals maize is the only major crop that contains appreciable amounts of carotenoids (Wurtzel, 2004). There are two generalized classes of carotenoids: carotenes and xanthophylls. Carotenoids are an important source of vitamin A, antioxidants and photo-protectants in plants (Goff and Klee, 2006). Antheraxanthin and violaxanthin, two xanthophylls are precursors of abscisic acid (ABA), which is essential for seed formation and induction of primary dormancy (Kermode, 2005). The carotenoid biosynthetic pathway is well studied and the enzymes involved in carotenogenesis have been documented in maize and other species (Giuliano et al., 2008; Li et al., 2009). In addition, association analyses of four key candidate genes involved in carotenoid biosynthesis revealed that natural polymorphisms at these loci are significantly associated with either carotenoid concentration or composition (Harjes et al., 2008; Yan et al., 2010; Zhou et al., 2012; Fu Z.Y. et al., 2013). Among these key candidate genes, *PSY1* experienced strong selection during maize domestication and improvement from white to yellow kernels, but after this selection bottleneck, there was very little sequence variation in *PSY1* within the yellow maize germplasm (Palaisa et al., 2004).

In the carotenoid biosynthetic pathway, violaxanthin de-epoxidase (VDE), a key enzyme of the xanthophyll cycle, has an important role in protecting the photosynthesis apparatus from damage, as excess light catalyzes the conversion of violaxanthin to zeaxanthin through antheraxanthin under high light. Zeaxanthin and antheraxanthin can transfer excess energy from chlorophyll, which is dissipated as heat and through scavenging reactive oxygen species, thus protecting the photosynthetic apparatus from photo damage (Eskling et al., 1997; Muller et al., 2001). In addition, lutein and zeaxanthin are associated with a reduced risk of cataract development and age-related macular degeneration (Abdel-Aal et al., 2013). VDE is a member of the lipocalin family (Bugos and Yamamoto, 1996) and has been isolated and purified from several plant species, such as romaine lettuce (Rockholm and Yamamoto, 1996) and spinach (Arvidsson et al., 1996), but has not yet been isolated from maize.

In this study, we cloned a gene encoding VDE, *ZmVDE1*, by a comparative genomic approach. Using Functional domain analysis of *ZmVDE1*, we confirmed that the VDE domain in *Z. mays* is highly consistent with the graminaceous plants. Furthermore, nucleotide diversity analysis and Tajima’s *D* test of *VDE1* in maize and teosinte indicated that a potential selective force had acted across the *ZmVDE1* locus. In addition, we identified a 4.2 Mb selective sweep with low recombination surrounding the *ZmVDE1* locus that resulted in severely reduced nucleotide diversity on chromosome 2.

## Materials and Methods

### Plant Materials and Phenotyping

A maize association panel consisting of 155 inbred lines (Yang et al., 2010) was used to detect associations between the nucleotide polymorphisms of *ZmVDE1* and carotenoid content as well as composition in maize kernels. Eighty-nine inbred lines from the 155 lines in the association panel and a set of 44 teosinte accessions from the CIMMYT gene bank (Supplementary Table S1) were used to resequence *ZmVDE1* in this study. The maize panel was planted in one-row plots with two replications in a randomized complete block design in Beijing, China during the summer of 2005, 2006, and 2007 and the winter of 2007 in Hainan, China. Each plot was 4 m long. Rows were spaced 0.67 m apart, and plants were grown at a planting density of 45,000 plants/ha; seeds were produced via self-pollination of each plant in the plot (Fu Z.Y. et al., 2013). The maize kernel carotenoids were measured after harvesting. The best linear unbiased predictors (BLUPs) for individual traits in each line were calculated using SAS 8.02 (SAS Institute, 1999) in the association mapping populations. BLUPs for each line across environments were used for the overall analysis (Fu Z.Y. et al., 2013).

### Candidate Gene Cloning

According to the zeaxanthin, antheraxanthin and violaxanthin inter conversion pathway (EMP, Enzymes and Metabolic Pathways database^1^), the protein sequence of the VDE homologous gene in *Arabidopsis thaliana, AVDE1*, was retrieved from TAIR^2^. The sequence homologous to *AVDE1* in maize was obtained via BLAST using AVDE1 protein sequence against the maize genomic sequence in the Maize Genetics Genomics Database (maizeGDB^3^). The related information for *ZmVDE1*, including nucleotide sequence, transcript and gene structure, was obtained from the Gramene database^4^. In addition, DNA motif was predicted using the PLACE^5^ and PlantCARE^6^ databases.

### Phylogenetic Tree Construction

To further investigate the genetic relationship of the *VDE* genes among different species, reference sequences were retrieved from the non-redundant protein sequence database using protein tool BLAST at NCBI^7^. And then the alignment file was constructed using ClustalW in MEGA 5.0. Phylogenetic tree was constructed using neighbor-joining criterion in MEGA 5.0 with 1,000 bootstrap tests for every node (Tamura et al., 2011). The uniform rates was applied for rates among sites. Gaps or missing data was treated as complete deletion.

### Quantification of Carotenoids

Multiple self-pollinated ears from each line for each replication were combined for high-performance liquid chromatography (HPLC) analysis. Measured metabolites included lutein, zeaxanthin, β-cryptoxanthin, α-carotene and β-carotene. Carotenoids were extracted as described in Chander et al. (2008) and quantified by standard regression against external standards (Kurilich and Juvik, 1999). External standard curves were constructed with eight serial dilutions and with three repeats for each dilution (*R*^2^≥ 0.99). The five carotenoids were separated on a reversed-phase C30 column (YMC Carotenoid, CT99S05-2546WT C30, 4.6 nm × 25 cm, 5 μm; Waters) at 30°C at 1.8 ml/min for the mobile phase, 75:20:5 (v/v/v) acetonitrile/methanol/dichloromethane, by scanning at 450 nm with a reference wave of 360 nm and were identified by the retention time of each standard. The peak times for lutein, zeaxanthin, β-cryptoxanthin, α-carotene and β-carotene were 8.37 min, 10.71, 19.94, 27.61, and 37.61 min, respectively. All phenotypic data were generated with ChemStation software (Agilent Technologies) (Xu et al., 2012).

### Genotyping and Sequence Analysis of *ZmVDE1*

Two primers that spanned most of the gene were used in conjunction with two additional sets of primers to sequence *ZmVDE1* in 89 maize lines and 44 teosinte entries (Supplementary Table S2). A 30-μl aliquot of the resulting PCR product from each line was sequenced directly using an ABI3730 sequencer. The sequences were assembled using ContigExpress in Vector NTI Advance 10 (Invitrogen), aligned using MUSCLE (Edgar, 2004) and manually corrected with BioEdit (Hall, 1999). Additionally, to determine the expression level of *ZmVDE1*, 557,955 polymorphic sites with a minor allele frequency (MAF) ≥0.05 and missing rate <25% were generated by RNA-Sequencing maize kernels collected 15 days after pollination (DAP) from 368 maize inbred lines (Fu J.J. et al., 2013). Using the expression levels of *ZmVDE1*, we looked for expression QTL and analyzed their correlations with carotenoid content. Subsequently, >120,000 single nucleotide polymorphisms (SNPs) distributed across chromosome 2 in the 368 maize lines were used for testing whether there were selective sweeps around *ZmVDE1*.

### Candidate Gene Association Mapping

The polymorphic sites including SNPs and insertions or deletions (InDels) of *ZmVDE1* with MAF ≥0.05 in 89 maize lines from the 155**-**line association panel were extracted using TASSEL 2.1 (Bradbury et al., 2007). Associations between the polymorphic sites and five carotenoid-related traits were carried out using a mixed linear model (Yu et al., 2006), which incorporated population structure and kinship (Yang et al., 2010) in TASSEL 2.1.

### Nucleotide Diversity and Tests for Selection

Nucleotide diversity (π) and Tajima’s *D* statistic were calculated for *ZmVDE1* and chromosome 2 using DNaSP version 5.0 (Librado and Rozas, 2009). The scaled per-nucleotide recombination parameter Rn (Hudson, 1987) is the length-weighted mean of Rn across sweep region estimated from 368 maize lines.

### Minimum Spanning Tree

The 89 maize inbred lines and 44 teosinte entries for the two subspecies *Z. mays* ssp. *mexicana* and *Z. mays* ssp. *parviglumis* (Supplementary Table S1) were used to construct a minimum spanning tree for *ZmVDE1*. Arlequin version 3.5 (Excoffier and Lischer, 2010) was used to calculate the minimum spanning tree nucleotide polymorphisms. Arlequin’s distance matrix output was used in Hapstar-0.6 (Teacher and Griffiths, 2011) to draw the minimum spanning tree.

## Results

### Cloning, Phylogenetic Analysis and Expression Pattern of *ZmVDE1*

A BLAST search with *AVDE1* protein sequences from *A. thaliana* (AT1G08550) against the maize genomic sequences in the maizeGDB database resulted in three hits for homologous genes, GRMZM2G027219 on chromosome 2, GRMZM2G701673 on chromosome 10 and GRMZM2G408706 on chromosome 8. The maize homologous gene GRMZM2G027219 had the highest identity with *AVDE1* (*E*-value = 4.734 × 10^-134^, 81.95% amino acid identity) and was referred to as *ZmVDE1*. *ZmVDE1* from maize inbred line B73 has five exons, and the full-length cDNA sequence is 1,761 base pairs (bp), encoding 446 amino acids. The length of the 5′untranslated region (UTR) and 3′UTR are 148 and 272 bp, respectively (**Supplementary Figure S1**). Motif Scan analysis (Sigrist et al., 2010) showed that the VDE domain (amino acid 163–360) is present in the mature protein of *ZmVDE1*. Using the PLACE and PlantCARE databases, we also found the following putative DNA motifs, which are probably involved in light responsiveness, in the regulatory region and introns of *ZmVDE1* (**Supplementary Figure S1**): I-box, G-box, GT-1 motif, TCT-motif, and MNF1-motif.

**FIGURE 1:**
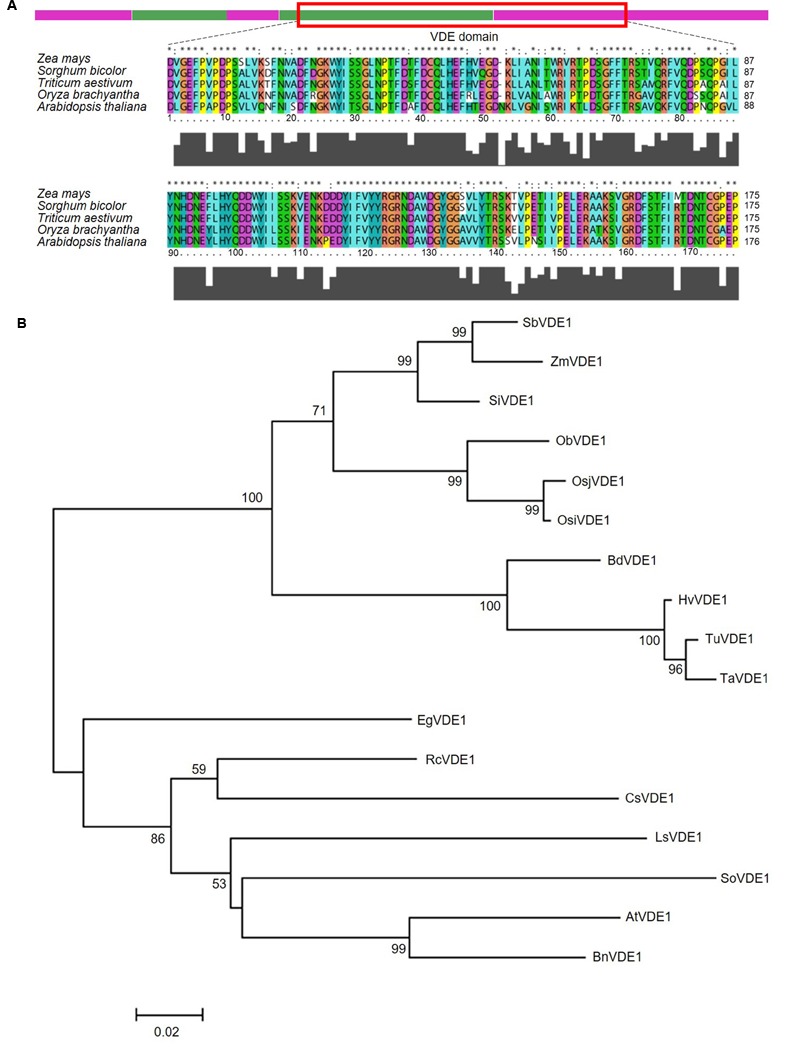
**Characterization of *ZmVDE1*. (A)** Schematic representation of the *ZmVDE1* protein and an amino acid alignment of the VDE domains from different plants. The VDE domain is indicated by a red box. Purple and green bars represent the five exons. Gray squares below the sequence indicate the extent of sequence similarity. **(B)** Phylogenetic tree of the VDE proteins. The tree was constructed on the basis of genes homologous with *ZmVDE1* by a neighbor-joining tree. Numbers above branches indicate the percentage of bootstrap values calculated from 1000 replicates. The scale bar gives a rough indication of sequence divergence. Sb, *Sorghum bicolor*; Zm, *Zea mays*; Si, *Setaria italic*; Ob, *Oryza brachyantha*; Osj, *Oryza sativa L. ssp. Japonica*; Osi, *Oryza sativa L. ssp. Indica*; Bd, *Brachypodium distachyon*; Hv, *Hordeum vulgare*; Tu, *Triticum urartu*; Ta, *Triticum aestivum*; Eg, *Elaeis guineensis*; Rc, *Ricinus communis*; Cs, *Cucumis sativus*; So, *Spinacia oleracea*; Ls, *Lactuca sativa*; At, *Arabidopsis thaliana*; Bn, *Brassica napus*.

Functional domain analysis of this genomic fragment in InterProScan and EBI QuickGO confirmed that the VDE domain in *Z. mays* is highly consistent with those of the graminaceous plants, such as *Triticum aestivum, Sorghum bicolor*, and *Oryza sativa* (**Figure 1A**). To further investigate the genetic relationship of the VDE proteins among different species, phylogenetic analysis of these homologs was conducted in graminaceous plants, herbaceous plants and oil crops (**Figure 1B**). *ZmVDE1* is grouped with all the graminaceous plant VDEs and is more closely related to the VDE of *Sorghum bicolor* than to other graminaceous crops. VDEs from other plants included oil crops and herbaceous plants in the another cluster.

Based on 53 samples ranging in developmental age from fertilization to maturity and consisting of the embryo, endosperm and whole-seed tissues from a previous study (Chen et al., 2014), we detected the expression pattern of *ZmVDE1* during maize kernel development. *ZmVDE1* was mainly expressed in the late stages of the embryo, endosperm and whole seed. Moreover, *ZmVDE1* at most time points was expressed at a higher level in the endosperm than in the embryo samples (**Supplementary Figure S2**). Additionally, Stelpflug et al. (2015) had enhanced their previously published microarray-based gene atlas of maize inbred line B73 (Sekhon et al., 2011, 2012) to include 79 distinct replicated samples that had been analyzed using RNA-sequencing. The expression pattern of *ZmVDE1* in the endosperm and whole-seed tissues was similar to the findings of Chen et al. (2014). Therefore, we inferred that *ZmVDE1* is active in the late stages of seed development, in accordance with its function as an important synthetase involved in nutrient accumulation in maize kernels.

### Association between *ZmVDE1* and Carotenoid-Related Traits in Maize Kernels

**FIGURE 2:**
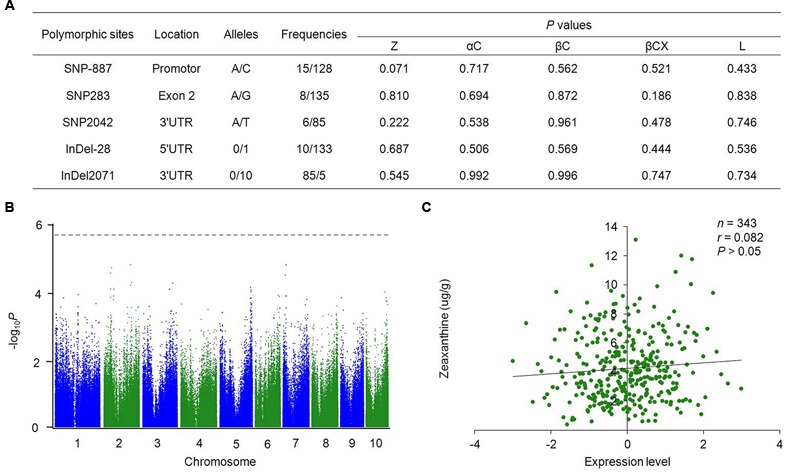
**Associations between *ZmVDE1* and carotenoid-related traits in maize kernels. (A)** Summary of significant polymorphisms from candidate gene–based association studies. The first base of the start codon is defined as position 1. All the *P* values were from a mixed linear model controlling for population structure and individual relatedness in TASSEL 2.1. Z, zeaxanthin; αC, α-carotene; βC, β-carotene; βCX, β-cryptoxanthin; L, lutein. **(B)** Manhattan plot of eQTL for *ZmVDE1*. The dashed horizontal line depicts the Bonferroni-adjusted significance threshold (1.8 × 10^-6^). eQTL, expression quantitative trait loci. **(C)** Plots of correlation between the level of *ZmVDE1* expression and zeaxanthin content in maize kernels. The *x* axis represents the normalized expression level of *ZmVDE1* in kernels collected at 15 DAP (Fu J.J. et al., 2013). The *y* axis represents the level of zeaxanthin. The *r* value is a Pearson correlation coefficient.

To investigate the nucleotide polymorphisms of *ZmVDE1* in maize, the full-length sequence including the promoter, 5′UTR, exons, 3′UTR and most of the introns was obtained from 89 inbred lines. Our re-sequencing results identified five nucleotide polymorphisms with MAF ≥0.05, two InDels and three SNPs, in a 3,724 bp genomic region (**Figure 2A**). On average, one in every 745 bp was polymorphic. Additionally, the distribution of the nucleotide diversity was not even across *ZmVDE1*. Among these polymorphic sites, only one SNP (SNP283) was located in the coding region, which affected residue 65 (which encodes a p.Val65Ile alteration) with no change in charge or hydrophobicity. The limited number of nucleotide polymorphisms within *ZmVDE1* across 89 maize inbred lines illustrates that *ZmVDE1* has an important role in the maize kernel with a conserved function.

Association analysis with a mixed linear model that controlled for both population structure and individual relatedness revealed that no polymorphic sites in *ZmVDE1* showed a significant association with any of the analyzed carotenoid-related traits at the *P* = 0.05 level (**Figure 2A**). Previous studies have shown the importance of transcript abundance in the control of carotenoid profiles (Aluru et al., 2008; Harjes et al., 2008; Bai et al., 2009). Thus, we tested the correlation between the polymorphisms identified in the DNA sequence and the *ZmVDE1* expression level to investigate whether *ZmVDE1* can regulate any carotenoid-related traits at the level of expression. At *P* < 1.8 × 10^-6^, there was no statistical correlation between DNA sequence polymorphisms of *ZmVDE1* and its expression level (**Figure 2B**). In addition, expression of *ZmVDE1* was not correlated with phenotypic variation for any measured carotenoid trait (**Figure 2C**, **Supplementary Figure S3**). Thus the limited natural variation of *ZmVDE1* makes no significant contribution to carotenoid variation.

### Nucleotide Diversity of *ZmVDE1* during Maize Domestication

**FIGURE 3:**
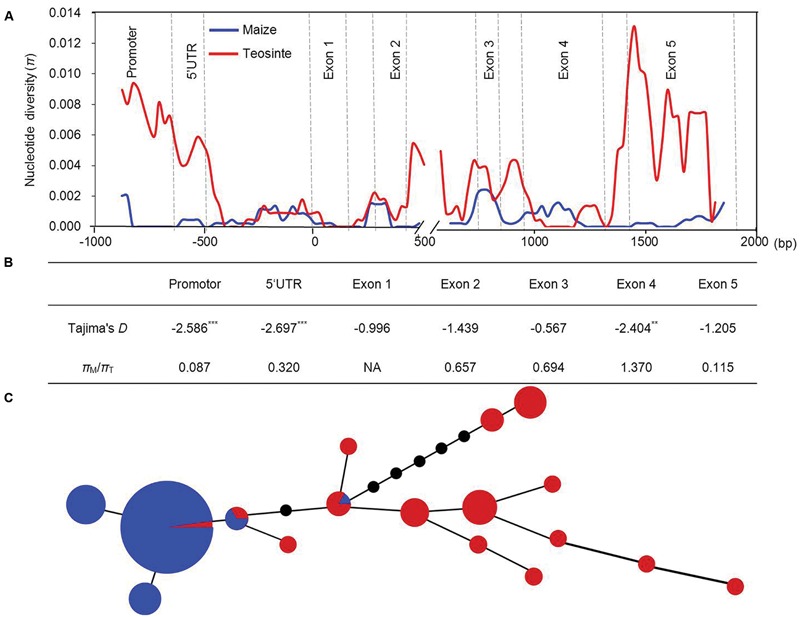
**Sequence diversity of the *ZmVDE1* locus between maize and teosinte. (A)** Nucleotide diversity revealed by comparisons between 89 maize lines and 44 teosinte entries across the *ZmVDE1* locus. Nucleotide diversity (π) for teosinte and maize was shown. **(B)** Tajima’s *D* test for non-neutral evolution and the ratios of *π* in maize (*π_M_*) to *π* in teosinte (*π_T_*) are shown. **P < 0.01; ***P < 0.001. **(C)** A minimum-spanning tree for *ZmVDE1* including 89 diverse maize sequences and 44 diverse teosinte sequences. Each haplotype group is represented by a circle with a color, and circle sizes represent the number of lines within the haplotype. Red and blue represent teosinte and maize, respectively.

To examine the levels of genetic diversity in *ZmVDE1*, we sequenced the promoter, 5′UTR, exons and introns of *ZmVDE1* in 44 teosinte entries consisting of two subspecies, Z*ea mays* ssp. *parviglumis* and *Zea mays* ssp. *mexicana* (Supplementary Table S1). Together with the *ZmVDE1* sequences from 89 maize lines, 15 polymorphic sites were identified (Supplementary Table S3). When we used a sliding window of 100 bp with a step size of 25 bp to calculate nucleotide diversity, we noted that nucleotide diversity was not equally distributed in either maize or teosinte, with exons containing less genetic variation (**Figure 3A**). In most parts of *ZmVDE1*, the average π over all maize lines was lower than that in teosinte, especially for the promoter and exon 5 regions, which had dramatically reduced diversity in maize (π*_M_/*π*_T_*= 0.087 and π*_M_*/π_T_= 0.115, respectively) (**Figure 3B**). This finding suggests that the promoter and exon 5 in *ZmVDE1* might have been under selection during the domestication of maize from teosinte.

To further investigate the evidence of selection during maize domestication, Tajima’s *D* test was conducted for the promoter, 5′UTR and exon regions of *ZmVDE1* in maize and teosinte. Tajima’s *D* test showed that promoter, 5′UTR and exon 4 regions of *ZmVDE1* had significantly negative parameter deviations (*P* < 0.01) (**Figure 3B**), which is consistent with directional selection (Jiao et al., 2012). The results suggest that a selective sweep might have occurred in those regions, leading to reduced nucleotide diversity across the *ZmVDE1* locus in maize.

### Evolution of the *ZmVDE1* Locus in Maize and Teosinte

**FIGURE 4:**
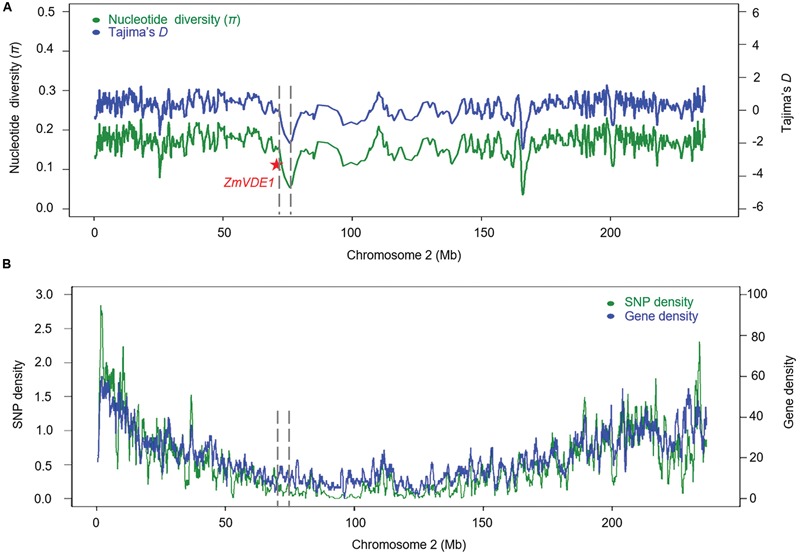
**Nucleotide variation on chromosome 2 in 368 maize inbred lines. (A)** Nucleotide diversity (*π*) and Tajima’s *D* for maize chromosome 2. The red star represents *ZmVDE1*. The dash line represents the selection sweep region of 4.2 Mb. **(B)** SNP density (green) and gene density (blue) calculated using a 1 Mb sliding window with a 100-kb step.

To further investigate the genetic signature of positive selection in the *ZmVDE1* region in more detail, we selected 120,204 high-quality SNPs on chromosome 2 with MAF ≥0.05 from a panel of 368 diverse maize inbred lines (Fu J.J. et al., 2013). Using a sliding window of 500 SNPs with a step size of 100 SNPs to calculate the nucleotide diversity and conduct Tajima’s *D* test, we found that the maize inbred lines had severely reduced nucleotide diversity (π = 0.123) across a 4.2 Mb region surrounding the *ZmVDE1* locus relative to other segments on chromosome 2 (**Figure 4A**). In addition, the statistic for Tajima’s *D* test was notably negative for a selective sweep in the 368 inbred lines, suggesting an excess of low-frequency polymorphisms relative to the expectation that might have resulted from the selection on the selective sweep in this population (**Figure 4A**). The large interval (4.2 Mb) associated with this selective sweep might be related to the low SNP or gene density and the recombination rate. As expected, no significant reduction in SNP or gene density was observed in the 4.2 Mb region surrounding the *ZmVDE1* locus. There are 478 SNPs in the 4.2 Mb selective sweep with one in every 8,786 bp being polymorphic, and there are 45 genes in the large interval, with one every 93.3 kb (**Figure 4B**). In contrast, there is one polymorphic nucleotide every 1,951 bp and one gene every 49.5 kb in other regions of chromosome 2 (**Figure 4B**). The nucleotide estimate for the population recombination rate indicated that the recombination rate across this sweep region (Rn = 0.0757) was 1.59-fold lower than it was at the known selection target *tga1* (Rn = 0.1205). These results suggested that few recombination events can be detected in this selective sweep because of the relatively low levels of polymorphism and that the low rate of recombination has contributed to the size of the sweep.

To gain deeper insights into the evolution of *ZmVDE1*, its full-length sequence including the promoter, 5′UTR, exons, 3′UTR and most of the introns from 89 Chinese elite inbred lines and 44 additional teosinte entries (Supplementary Table S1) was used to construct a minimum-spanning tree. In total, we identified five haplotypes for the 89 diverse maize lines, of which Hap1 predominated, and 15 haplotypes for 44 teosinte entries (Supplementary Table S4). With these haplotypes, a minimum-spanning tree was built (**Figure 3C**). The 17 haplotypes formed two distinguishable clusters: a maize haplotype cluster and a teosinte haplotype cluster. The maize haplotype cluster contained almost all the maize lines and two teosinte entries, whereas the teosinte haplotype cluster was composed of teosinte entries with one additional maize line. These results revealed that *VDE1* was domesticated with fewer haplotypes in maize comparing with which in teosinte. (**Figures 3A,B**), the nucleotide diversity was much higher in teosinte than in maize.

## Discussion

Violaxanthin de-epoxidase carries out an important role in the photo-protective xanthophyll cycle by catalyzing the formation of non–provitamin A carotenoids such as zeaxanthin that function during the strong light response (Rockholm and Yamamoto, 1996; Li et al., 2009). However, important questions about whether the expression of this protein was selected for during the domestication of maize are still unanswered. In this study, we used association analysis, nucleotide diversity detection and evolutionary analyses to confirm the significance of *ZmVDE1* in maize domestication.

### VDE Has a Conserved Function in Plants

In maize, *ZmVDE1* contains five putative circadian DNA motifs, I-box (GATA motif), G-box, GT-1 motif, TCT-motif and MNF1-motif (**Supplementary Figure S1**). These motifs are thought to be involved in light regulation and circadian rhythm expression (Borello et al., 1993; Piechulla et al., 1998; Xu et al., 2003; Kidokoro et al., 2009). In addition to the light-regulated motifs, the other most important motifs in introns were *cis*-acting elements involved in plant responses to environmental stresses, including MYB2CONSENSUSAT(YAACKG), CBFHV (RYCGAC), RAV1AAT (CAACA), CCAATBOX1 (CCAAT), WBOXATNPR1(TTGAC), GT1GMSCAM4(GAAAAA), MYB1 AT (WAACCA), MYBCORE (CNGTTR), MYCCONSENSUSAT (CANNTG) and WRKY71OS (TGAC), which indicated that the expression of *ZmVDE1* might also be regulated by transcription factors related to stress tolerance. All of these motifs showed high identities with other gramineous VDEs such as those from *T. aestivum* and *Oryza sativa*, especially in the regions corresponding to the mature proteins (Gao et al., 2013).

Violaxanthin de-epoxidase has a conserved function converting violaxanthin to antheraxanthin and then to zeaxanthin in the xanthophyll cycle, which is a photo-protection mechanism conserved among photosynthetic plants and algae to protect the photosynthetic apparatus from excess light (North et al., 2005). Meanwhile, downstream of the xanthophyll cycle, violaxanthin and zeaxanthin are the intermediate products in the biosynthesis pathway of ABA, which regulates the development process, seed maturation, stomatal closure and stress responses (Finkelstein et al., 2002; Zhu, 2002; Wang and Song, 2008). Therefore, *VDE* and zeaxanthin epoxidase (*ZEP*) have been the targets of genetic manipulation to better understand the carotenoid synthetic pathway. Römera et al. (2002) found that the amount of violaxanthin was diminished dramatically, and in some cases the amount of the monoepoxy intermediate antheraxanthin was increased, as a consequence of the genetic manipulation of transformation with sense and antisense constructs encoding zeaxanthin epoxidase, even though the total carotenoid content and ABA content were both increased. Thus *VDE* might regulate the synthesis of ABA (Owen et al., 1992; Marin et al., 1996; Pogson et al., 1996; Audran et al., 1998). The enhanced activity of VDE under excess light could reduce the content of ABA, perhaps by acting on the xanthophyll cycle and regulating the synthesis of the ABA precursor.

### Limited Natural Variations within *ZmVDE1* Are Not Associated with Carotenoid-Related Traits

Candidate gene association mapping in DNA from kernels grown in multiple environments indicated that none of the five polymorphisms in *ZmVDE1* were significantly associated with carotenoid-related traits in maize kernels. To confirm this result, we also extracted 220 SNPs of *ZmVDE1* in 368 maize inbred lines (Fu J.J. et al., 2013) to perform a regional association analysis for carotenoid-related traits. No significant SNPs were identified, consistent with the association analysis in the 89 maize inbred lines. In addition, *ZmVDE1* expression was not correlated with any of the measured carotenoid-related traits. One possible explanation for the absence of an association may be the small size of our association panel. It is well known that association mapping studies may be adversely affected by two major interconnected factors, the dominance of a SNP with a lower MAF and a small population size (Manolio et al., 2009). Simulation results of the effect on QTL detection power with increasing population size revealed that a population with 500 lines provides an 80% probability of detecting a gene that explains 3% or more of the phenotypic variation (Yan et al., 2011). So some important genetic variations may not have been detected in our association panel with its relatively small population size. Another explanation for this absence of an association is that *ZmVDE1* has an important and conserved role in plants. Additionally, *ZmVDE1* was expressed throughout the leaf with highest expression level at maturing and mature zones (**Supplementary Figure S4**). Moreover, expression of *ZmVDE1* in seedling leaves was statistically higher than that in maize kernels (Chen et al., 2014; Stelpflug et al., 2015), similar to that of *AtVDE* in *A. thaliana* (North et al., 2005), suggesting that *ZmVDE1* also has an important protective role in chloroplasts (Audran et al., 2001).

Previous studies have shown that association mapping results of candidate genes cloned by homology-based cloning is quite different among maize. For example, *ZmGS3* and *ZmGW2* have co-orthologous relationships with the rice region containing *GS3* and *GW2*, respectively. *ZmGS3* was slightly associated with maize kernel length and one-hundred kernel weight (HKW), with the *P*-values ranging between 0.05 and 0.01, whereas *ZmGW2* was significantly associated with kernel size (*P* < 10^-3^) (Li et al., 2010a,b). These results for *ZmVDE1, ZmGS3* and *ZmGW2* imply that the power of an association study is dependent on various factors, such as the gene effect, the genetic architecture of the quantitative trait and the size of association population and so on. So, in addition to the association analysis, we still need other genetic methods to validate gene function.

### *ZmVDE1* Has Been under Strong Selection during Maize Domestication

Because of the role of the VDE in the xanthophyll cycle, an important photo-protection mechanism, it is easy to understand why *ZmVDE1* was a target of the domestication or improvement process in maize. In our study, *ZmVDE1* was less diverse at the nucleotide level in maize than in teosinte, especially in the promoter and 5′UTR regions, consistent with a significantly negative Tajima’s *D* parameter, which may indicate that this region has undergone a recent and strong positive selection. While, significant reductions in diversity were detected in the VDE domain region (*π_M_/π_T_* = 0.079) with negative Tajima’s *D* parameter (Tajima’s *D* = -1.576), which may be because that VDE domain variants within these regions might have experienced positive selection during maize domestication and improvement. After this selective force on *ZmVDE1*, the VDE domain became fixed with no polymorphic sites, as indicated by widespread Chinese maize inbred lines (**Figure 2A**).

Positive directional selection results in reduced variability in particular regions, which are referred to as selective sweep regions (Tian et al., 2009). According to our results, *ZmVDE1* is located in a genomic region of 4.2 Mb that was affected by a selective sweep based on the 368**-**line maize association population. A larger selected region (5.2 Mb) located on chromosome 2 has been reported, and there was a strong signal for *ZmVDE1* in this region (Hufford et al., 2012), which supports our findings. Similar evidence for a domestication-related selection sweep has also been reported in maize. A selective sweep extending 60–90 kb upstream of *tb1* indicated that *tb1* had a role in the domestication of maize from teosinte between 6,000 and 10,000 years ago (Clark et al., 2004). Maize *PSY1* on chromosome 6, which controls kernel color, has undergone very strong selection for the yellow kernel color phenotype. Patterns of diversity in this region are consistent with the occurrence of a large selective sweep (>0.5 Mb) at *PSY1* in maize breeding lines (Palaisa et al., 2004). Maize also shows severely reduced nucleotide diversity relative to teosinte across a large selective sweep (1.1 Mb) on chromosome 10 that has been the target of strong selection during maize domestication (Tian et al., 2009). In addition, a study on chromosome 3, which contains the largest selected region (2.2 Mb), using 278 temperate maize inbred lines from different stages of breeding history showed that modern breeding has introduced highly dynamic genetic changes into the maize genome (Jiao et al., 2012). At the same time, artificial selection leads to a reduction in nucleotide diversity and an increase in the proportion of rare alleles (Jiao et al., 2012). Collectively, these results imply that *ZmVDE1* is a major domestication- and improvement-related gene involved in the carotenoid biosynthesis pathway. The challenge for the future is to figure out how the evolution and selection of this gene have occurred.

## Author Contributions

JX analyzed the data and wrote the manuscript, HY and ZL performed the experiments, XY designed the study, CC advised on the study and manuscript, HL analyzed the data and wrote the manuscript.

## Conflict of Interest Statement

The authors declare that the research was conducted in the absence of any commercial or financial relationships that could be construed as a potential conflict of interest.

## Footnotes

1 http://www.plantcyc.org

2 http://www.arabidopsis.org

3 http://www.maizegdb.org

4 http://www.gramene.org/

5 http://ppdb.agr.gifu-u.ac.jp/ppdb/cgi-bin/index.cgi

6 http://bioinformatics.psb.ugent.be/webtools/plantcare/html/

7 http://www.ncbi.nlm.nih.gov

## Supplementary Material

FIGURE S1 ***ZmVDE1* gene structure based on the genomic sequence from maize inbred line B73.** The red, sky blue, and yellow represent the transcription start and termination position, presumptive motif and coding sequence of *ZmVDE1*.

FIGURE S2 **Expression patterns of *ZmVDE1* in various tissues at different developmental stages in maize line B73.** FPKM (red; Stelpflug et al., 2015) and relative expression (blue; Chen et al., 2014) represent the expression level of *ZmVDE1* based on these two methods. **(A–C)** Expression of *ZmVDE1* in seed **(A)**, endosperm **(B)** and embryo **(C)**. FPKM, fragments per kilobase of transcript per million mapped reads.

FIGURE S3 **Plots of correlation between *ZmVDE1* expression and carotenoid-related traits in maize kernels. (A–D)** The *x* axis represents the normalized expression of *ZmVDE1* in kernels collected at 15 DAP. The *y* axis represents the level of lutein **(A)**, β-cryptoxanthin **(B)**, α-carotene **(C)** and β-carotene **(D)**. The *r* value is a Pearson correlation coefficient.

FIGURE S4 **Expression patterns of *ZmVDE1* in maize leaf in maize line B73.** Histogram represent the expression level of *ZmVDE1* based on Chen et al. (2014).

## References

[B1] Abdel-Aal E. M. Akhtar H. Zaheer K. Ali R. (2013). Dietary sources of lutein and zeaxanthin carotenoids and their role in eye health. *Nutrients* 5 1169–1185. 10.3390/nu5041169 23571649 PMC3705341

[B2] Aluru M. Xu Y. Guo R. Wang Z. G. Li S. S. White W. (2008). Generation of transgenic maize with enhanced provitamin A content. *J. Exp. Bot.* 59 3551–3562. 10.1093/jxb/ern212 18723758 PMC2561147

[B3] Arvidsson P. O. Bratt C. E. Carlsson M. Akerlund H. E. (1996). Purification and identification of the violaxanthinde-epoxidase as a 43 kDa protein. *Photosynth. Res.* 49 119–129. 10.1007/BF00117662 24271609

[B4] Audran C. Borel C. Frey A. Sotta B. Meyer C. Simonneau T. (1998). Expression studies of the zeaxanthin epoxidase gene in *Nicotiana plumbaginifolia*. *Plant Physiol.* 118 1021–1028. 10.1104/pp.118.3.1021 9808747 PMC34775

[B5] Audran C. Liotenberg S. Gonneau M. North H. Frey A. Tap-Waksman K. (2001). Localisation and expression of zeaxanthine poxidase mRNA in *Arabidopsis* in response to drought stress and during seed development. *Aust. J. Plant Physiol.* 28 1161–1173. 10.1071/PP00134

[B6] Bai L. Kim E. H. DellaPenna D. Brutnell T. P. (2009). Novel lycopene epsilon cyclase activities in maize revealed through perturbation of carotenoid biosynthesis. *Plant J.* 59 588–599. 10.1111/j.1365-313X.2009.03899.x 19392686

[B7] Borello U. Ceccarelli E. Giuliano G. (1993). Constitutive, light-responsive and circadian clock-responsive factors compete for the different l box elements in plant light-regulated promoters. *Plant J.* 4 611–619. 10.1046/j.1365-313X.1993.04040611.x 8252065

[B8] Bradbury P. J. Zhang Z. Kroon D. E. Casstevens T. M. Ramdoss Y. Buckler E. S. (2007). TASSEL: software for association mapping of complex traits in diverse samples. *Bioinformatics* 23 2633–2635. 10.1093/bioinformatics/btm308 17586829

[B9] Bugos R. C. Yamamoto H. Y. (1996). Molecular cloning of violaxanthinde-epoxidase from romaine lettuce and expression in *Escherichia coli*. *Proc. Natl. Acad. Sci. U.S.A.* 93 6320–6325. 10.1073/pnas.93.13.6320 8692813 PMC39020

[B10] Chander S. Meng Y. J. Zhang Y. R. Yan J. B. Li J. S. (2008). Comparison of nutritional traits variability in selected eighty-seven inbreds from Chinese maize (*Zea mays* L.) germplasm. *J. Agric. Food Chem.* 56 6506–6511. 10.1021/jf7037967 18620402

[B11] Chen J. Zeng B. Zhang M. Xie S. J. Wang G. K. Hauck A. (2014). Dynamic transcriptome landscape of maize embryo and endosperm development. *Plant Physiol.* 166 252–264. 10.1104/pp.114.240689 25037214 PMC4149711

[B12] Clark R. M. Linton E. Messing J. Doebley J. F. (2004). Pattern of diversity in the genomic region near the maize domestication gene tb1. *Proc. Natl. Acad. Sci. U.S.A.* 101 700–707. 10.1073/pnas.2237049100 14701910 PMC321743

[B13] Doebley J. Stec A. (1991). Genetic analysis of the morphological differences between maize and teosinte. *Genetics* 129 285–295. 1682215 10.1093/genetics/129.1.285 PMC1204577

[B14] Doebley J. Stec A. Gustus C. (1995). Teosinte branched1 and the origin of maize: evidence for epistasis and the evolution of dominance. *Genetics* 141 333–346. 8536981 10.1093/genetics/141.1.333 PMC1206731

[B15] Doebley J. Stec A. Hubbard L. (1997). The evolution of apical dominance in maize. *Nature* 386 485–488. 10.1038/386485a0 9087405

[B16] Doebley J. Stec A. Wendel J. Edwards M. (1990). Genetic and morphological analysis of a maize-teosinte F2 population: implications for the origin of maize. *Proc. Natl. Acad. Sci. U.S.A.* 87 9888–9892. 10.1073/pnas.87.24.9888 11607138 PMC55279

[B17] Dorweiler J. Stec A. Kermicle J. Doebley J. (1993). Teosinte glume architecture 1: a genetic locus controlling a key step in maize evolution. *Science* 262 233–235. 10.1126/science.262.5131.233 17841871

[B18] Edgar R. C. (2004). MUSCLE: multiple sequence alignment with high accuracy and high throughput. *Nucleic Acids Res.* 32 1792–1797. 10.1093/nar/gkh340 15034147 PMC390337

[B19] Eskling M. Arvidsson P. O. Akerlund H. E. (1997). The xanthophyll cycle, its regulation and components. *Physiol. Plant.* 100 806–816. 10.1111/j.1399-3054.1997.tb00007.x

[B20] Excoffier L. Lischer H. E. (2010). Arlequin suite ver 3.5: a new series of programs to perform population genetics analyses under Linux and Windows. *Mol. Ecol. Resour.* 10 564–567. 10.1111/j.1755-0998.2010.02847.x 21565059

[B21] Finkelstein R. R. Gampala S. S. Rock C. D. (2002). Abscisic acid signaling in seeds and seedlings. *Plant Cell* 14 S15–S45. 10.1105/tpc.010441 12045268 PMC151246

[B22] Fu J. J. Cheng Y. B. Linghu J. J. Yang X. H. Kang L. Zhang Z. X. (2013). RNA sequencing reveals the complex regulatory network in the maize kernel. *Nat. Commun.* 4 2832–2844. 10.1038/ncomms3832 24343161

[B23] Fu Z. Y. Chai Y. C. Zhou Y. Yang X. H. Warburton M. L. Xu S. T. (2013). Natural variation in the sequence of PSY1 and frequency of favorable polymorphisms among tropical and temperate maize germplasm. *Theor. Appl. Genet.* 126 923–935. 10.1007/s00122-012-2026-0 23238762

[B24] Gao Z. M. Liu Q. Zheng B. Chen Y. (2013). Molecular characterization and primary functional analysis of PeVDE, a violaxanthin de-epoxidase gene from bamboo (*Phyllostachys edulis*). *Plant Cell Rep.* 32 1381–1391. 10.1007/s00299-013-1450-1 23640082

[B25] Giuliano G. Tavazza R. Diretto G. Beyer P. Taylor M. A. (2008). Metabolic engineering of carotenoid biosynthesis in plants. *Trends Biotechnol.* 26 139–145. 10.1016/j.tibtech.2007.12.003 18222560

[B26] Goff S. A. Klee H. J. (2006). Plant volatile compounds: sensory cues for health and nutritional value? *Science* 311 815–819. 10.1126/science.1118446 16469919

[B27] Hall T. A. (1999). BioEdit: a user-friendly biological sequence alignment editor and analysis program for Windows 95/98/NT. *Nucl. Acids Symp. Ser.* 41 95–98.

[B28] Harjes C. E. Rocheford T. R. Bai L. Brutnell T. P. Kandianis C. B. Sowinski S. G. (2008). Natural genetic variation in lycopene epsilon cyclase tapped for maize biofortification. *Science* 319 330–333. 10.1126/science.1150255 18202289 PMC2933658

[B29] Hudson R. R. (1987). Estimating the recombination parameter of a finite population model without selection. *Genet. Res.* 50 245–250. 10.1017/S0016672300023776 3443297

[B30] Hufford M. B. Xu X. Heerwaarden J. Pyhäjärvi T. Chia J. M. Cartwright R. A. (2012). Comparative population genomics of maize domestication and improvement. *Nat. Genet.* 44 808–811. 10.1038/ng.2309 22660546 PMC5531767

[B31] Institute S. A. S. (1999). *SAS Software.* Cary, NC: SAS Institute.

[B32] Jiao Y. P. Zhao H. N. Ren L. H. Song W. B. Zeng B. Guo J. J. (2012). Genome-wide genetic changes during modern breeding of maize. *Nat. Genet.* 44 812–815. 10.1038/ng.2312 22660547

[B33] Kermode A. R. (2005). Role of abscisic acid in seed dormancy. *J. Plant Growth Regul.* 24 319–344. 10.1007/s00344-005-0110-2

[B34] Kidokoro S. Maruyama K. Nakashima K. Imura Y. Narusaka Y. Shinwari Z. K. (2009). The phytochrome-Interacting factor PIF7 negatively regulates DREB1 expression under circadian control in *Arabidopsis*. *Plant Physiol.* 151 2046–2057. 10.1104/pp.109.147033 19837816 PMC2785984

[B35] Kurilich A. C. Juvik J. A. (1999). Simultaneous quantification of carotenoids and tocopherols in corn kernel extracts by HPLC. *J. Liq. Chromatogr. Relat. Technol.* 22 2925–2934. 10.1081/JLC-100102068

[B36] Li F. Tsfadia O. Wurtzel E. T. Tzfadia O. Wurtzel E. T. (2009). The phytoene synthase gene family in the Grasses: subfunctionalization provides tissue-specific control of carotenogenesis. *Plant Signal. Behav.* 4 208–211. 10.4161/psb.4.3.7798 19721751 PMC2652530

[B37] Li Q. Li L. Yang X. H. Warburton M. L. Bai G. H. Dai J. R. (2010a). Relationship, evolutionary fate and function of two maize co-orthologs of rice GW2 associated with kernel size and weight. *BMC Plant Biol.* 10:143 10.1186/1471-2229-10-143 PMC3017803 20626916

[B38] Li Q. Yang X. H. Bai G. H. Warburton M. L. Mahuku G. Gore M. (2010b). Cloning and characterization of a putative GS3 ortholog involved in maize kernel development. *Theor. Appl. Genet.* 120 753–763. 10.1007/s00122-009-1196-x 19898828

[B39] Librado P. Rozas J. (2009). DnaSP v5: a software for comprehensive analysis of DNA polymorphism data. *Bioinformatics* 25 1451–1452. 10.1093/bioinformatics/btp187 19346325

[B40] Manolio T. A. Collins F. S. Cox N. J. Goldstein D. B. Hindorff L. A. Hunter D. J. (2009). Finding the missing heritability of complex diseases. *Nature* 461 747–753. 10.1038/nature08494 19812666 PMC2831613

[B41] Marin E. Nussaume L. Quesada A. Gonneau M. Sotta B. Hugueney P. (1996). Molecular identification of zeaxanthin epoxidase of *Nicotiana plumbaginifolia*, a gene involved in abscisic acid biosynthesis and corresponding to the ABA locus of *Arabidopsis thaliana*. *EMBO J.* 15 2331–2342. 8665840 PMC450162

[B42] Muller K. R. Mika S. Ratsch G. Tsuda K. Scholkopf B. (2001). An introduction to kernel-based learning algorithms. *IEEE Trans. Neural Netw.* 12 181–201. 10.1109/72.914517 18244377

[B43] North H. M. Frey A. Boutin J. P. Sotta B. Marion-Poll A. (2005). Analysis of xanthophyll cycle gene expression during the adaptation of *Arabidopsis* to excess light and drought stress: changes in RNA steady-state levels do not contribute to shortterm responses. *Plant Sci.* 169 115–124. 10.1016/j.plantsci.2005.03.002

[B44] Owen M. Gandecha A. Cockburn B. Whitelam G. (1992). Synthesis of a functional anti-phytochrome single-chain Fv protein in transgenic tobacco. *Biotechnology (N. Y.)* 10 790–794. 10.1038/nbt0792-790 1368269

[B45] Palaisa K. Morgante M. Tingey S. Rafalski A. (2004). Long-range patterns of diversity and linkage disequilibrium surrounding the maize Y1 gene are indicative of an asymmetric selective sweep. *Proc. Natl. Acad. Sci. U.S.A.* 101 9885–9890. 10.1073/pnas.0307839101 15161968 PMC470768

[B46] Piechulla B. Merforth N. Rudolph B. (1998). Identification of tomato Lhc promoter regions necessary for circadian expression. *Plant Mol. Biol.* 38 655–662. 10.1023/A:1006094015513 9747810

[B47] Piperno D. R. Ranere A. J. Holst I. Iriarte J. Dickau R. (2009). Starch grain and phytolith evidence for early ninth millennium B.P. maize from the Central Balsas River Valley, Mexico. *Proc. Natl. Acad. Sci. U.S.A.* 106 5019–5024. 10.1073/pnas.0812525106 19307570 PMC2664021

[B48] Pogson B. McDonald K. A. Truong M. Britton G. DellaPenna D. (1996). *Arabidopsis* carotenoid mutants demonstrate that lutein is not essential for photosynthesis in higher plants. *Plant Cell* 8 1627–1639. 10.1105/tpc.8.9.1627 8837513 PMC161303

[B49] Rockholm D. C. Yamamoto H. Y. (1996). Violaxanthin De-Epoxidase: purification of a 43-Kilodalton lumenal protein from lettuce by lipid-affinity precipitation with Monogalactosyldiacylglyceride. *Plant Physiol.* 110 697–703. 10.1104/pp.110.2.697 8742341 PMC157766

[B50] Römera S. Lübeckb J. Kaudera F. Steigerc S. Adomata C. Sandmannc G. (2002). Genetic engineering of a zeaxanthin-rich potato by antisense inactivation and co-suppression of carotenoid epoxidation. *Metab. Eng.* 4 263–272. 10.1006/mben.2002.0234 12646321

[B51] Sekhon R. S. Childs K. L. Santoro N. Foster C. Buell C. R. Leon N. (2012). Transcriptional and metabolic analysis of senescence induced by preventing pollination in maize. *Plant Physiol.* 159 1730–1744. 10.1104/pp.112.199224 22732243 PMC3425209

[B52] Sekhon R. S. Lin H. N. Childs K. L. Hansey C. N. Buell C. R. Leon N. (2011). Genome-wide atlas of transcription during maize development. *Plant J.* 66 553–563. 10.1111/j.1365-313X.2011.04527.x 21299659

[B53] Sigrist C. A. Cerutti L. Castro E. Langendijk-Genevaux P. S. Bulliard V. Bairoch A. (2010). PROSITE, a protein domain database for functional characterization and annotation. *Nucleic Acids Res.* 38 D161–D166. 10.1093/nar/gkp885 19858104 PMC2808866

[B54] Stelpflug S. C. Sekhon R. S. Vaillancourt B. Hirsch C. N. Buell C. R. Leon N. (2015). An expanded maize gene expression atlas based on RNA-sequencing and its use to explore root development. *Plant Genome* 9 10.3835/plantgenome2015.04.0025 27898762

[B55] Studer A. Zhao Q. Ibarra J. R. Doebley J. (2011). Identification of a functional transposon insertion in the maize domestication gene tb1. *Nat. Genet.* 43 1160–1163. 10.1038/ng.942 21946354 PMC3686474

[B56] Tamura K. Peterson D. Peterson N. Stecher G. Nei M. Kumar S. (2011). MEGA5: molecular evolutionary genetics analysis using maximum likelihood, evolutionary distance, and maximum parsimony methods. *Mol. Biol. Evol.* 28 2731–2739. 10.1093/molbev/msr121 21546353 PMC3203626

[B57] Teacher A. G. Griffiths D. J. (2011). HapStar: automated haplotype network layout and visualization. *Mol. Ecol. Resour.* 11 151–153. 10.1111/j.1755-0998.2010.02890.x 21429113

[B58] Tian F. Stevens N. M. Buckler Iv E. S. (2009). Tracking footprints of maize domestication and evidence for a massive selective sweep on chromosome 10. *Proc. Natl. Acad. Sci. U.S.A.* 106 9979–9986. 10.1073/pnas.0901122106 19528660 PMC2702805

[B59] van Heerwaarden J. Doebley J. Briggs W. H. Glaubitz J. C. Goodman M. M. Gonzalez J. J. S. (2011). Genetic signals of origin, spread, and introgression in a large sample of maize landraces. *Proc. Natl. Acad. Sci. U.S.A.* 108 1088–1092. 10.1073/pnas.1013011108 21189301 PMC3024656

[B60] Wang H. Nussbaum-Wagler T. Li B. L. Zhao Q. Vigouroux Y. Faller M. (2005). The origin of the naked grains of maize. *Nature* 386 485–488. 10.1038/nature03863 PMC1464477 16079849

[B61] Wang P. T. Song C. P. (2008). Guard-cell signaling for hydrogen peroxide and abscisic acid. *New Phytol.* 178 703–718. 10.1111/j.1469-8137.2008.02431.x 18373649

[B62] Wang R. L. Stec A. Hey J. Lukens L. Doebley J. (1999). The limits of selection during maize domestication. *Nature* 398 236–239. 10.1038/18435 10094045

[B63] Whitt S. R. Wilson L. M. Tenaillon M. L. Gaut B. S. Buckler E. S. (2002). Genetic diversity and selection in the maize starch pathway. *Proc. Natl. Acad. Sci. U.S.A.* 99 12959–12962. 10.1073/pnas.202476999 12244216 PMC130568

[B64] Wurtzel E. T. (2004). Genomics, genetics, and biochemistry of maize carotenoid biosynthesis. *Recent Adv. Phytochem.* 38 85–110. 10.1016/S0079-9920(04)80006-6

[B65] Xu S. T. Zhang D. L. Cai Y. Zhou Y. Trushar S. Farhan A. (2012). Dissecting tocopherols content in maize (*Zea mays* L.), using two segregating populations and high-density single nucleotide polymorphism markers. *BMC Plant Biol.* 12:201 10.1186/1471-2229-12-201 PMC3502391 23122295

[B66] Xu Z. F. Chye M. L. Li H. Y. Xu F. X. Yao K. M. (2003). G-box binding coincides with increased *Solanum melongena* cysteine proteinase expression in senescent fruits and circadian-regulated leaves. *Plant Mol. Biol.* 51 9–19. 10.1023/A:1020859518877 12602887

[B67] Yan J. B. Kandianis C. B. Harjes C. E. Bai L. Kim E. H. Yang X. H. (2010). Rare genetic variation at *Zea mays* crtRB1 increases β-carotene in maize grain. *Nat. Genet.* 42 322–327. 10.1038/ng.551 20305664

[B68] Yan J. B. Warburton M. Crouch J. (2011). Association mapping for enhancing maize (*Zea mays* L.) genetic improvement. *Crop Sci.* 51 433–449. 10.2135/cropsci2010.04.0233

[B69] Yang X. H. Yan J. B. Shah T. Warburton M. L. Li Q. Li L. (2010). Genetic analysis and characterization of a new maize association mapping panel for quantitative trait loci dissection. *Theor. Appl. Genet.* 121 417–431. 10.1007/s00122-010-1320-y 20349034

[B70] Yu J. M. Pressoir G. Briggs W. H. Bi I. V. Yamasaki M. Doebley J. F. (2006). A unified mixed-model method for association mapping that accounts for multiple levels of relatedness. *Nat. Genet.* 38 203–208. 10.1038/ng1702 16380716

[B71] Zhou Y. Han Y. J. Li Z. G. Fu Y. Fu Z. Y. Xu S. T. (2012). ZmcrtRB3 encodes a carotenoid hydroxylase that affects the accumulation of α-carotene in maize kernel. *J. Integr. Plant Biol.* 54 260–269. 10.1111/j.1744-7909.2012.01106.x 22348777

[B72] Zhu J. K. (2002). Salt and drought stress signal transduction in plants. *Annu. Rev. Plant Biol.* 53 247–273. 10.1146/annurev.arplant.53.091401.143329 12221975 PMC3128348

