# Supplementary material for: Genetic Diversity and Molecular Evolution of a Violaxanthin De-epoxidase Gene in Maize
Source: Front Genet. 2016 Jul 26;7:131. doi: 10.3389/fgene.2016.00131 (PMC4960258; doi:10.3389/fgene.2016.00131)
Supplement: Supplementary file 2 [file Table_2.DOC]

**TABLE S2** List of primers used in this study.

| Name^a^ | Sequence (5′–3′) |
| --- | --- |
| *ZmVDE1*_1F | GGCTGAAACGAACACGC |
| *ZmVDE1*_1R | AGAATTACACTCGGCTACGGT |
| *ZmVDE1*_2F | TGCCACCGTAGCCGAGTG |
| *ZmVDE1*_2R | TGGGTCTAGTGTATCCTGTCCG |
| *TeoVDE1*_3F | GGGCTGCTTGGTTCGG |
| *TeoVDE1*_3R | TGCTCAACTTCAGTCGCTTC |

^a^Primers *ZmVDE1*_1F/1R and *ZmVDE1*_2F/2R were used to sequence the 5'UTR, exons, 3'UTR and most of the introns of *ZmVDE1* in the 89 maize lines. Primers *ZmVDE1*_1F/1R and *TeoVDE1*_3F/3R were used to sequence the 5'UTR, exons and most of the introns of *ZmVDE1* in the 44 teosinte entries.
