# Supplementary material for: Genetic Diversity and Molecular Evolution of a Violaxanthin De-epoxidase Gene in Maize
Source: Front Genet. 2016 Jul 26;7:131. doi: 10.3389/fgene.2016.00131 (PMC4960258; doi:10.3389/fgene.2016.00131)
Supplement: Supplementary file 3 [file Table_3.DOC]

**TABLE S3** Summary of *ZmVDE1* polymorphic sites in 89 maize inbred lines and 44 teosinte entries.

| Polymorphism location | Total | SNPs | InDels |
| --- | --- | --- | --- |
| Promoter | 2 | 2 | 0 |
| 5’UTR | 1 | 0 | 1 |
| Exons^a^ | 9 | 9 | 0 |
| Introns | 3 | 3 | 0 |
| 3'UTR | 0 | 0 | 0 |

^a^Eight of these SNPs resulted in synonymous amino acid substitutions, whereas one resulted in a non-synonymous substitution which from Val to Ile alteration.
