# Supplementary material for: Genetic Diversity and Molecular Evolution of a Violaxanthin De-epoxidase Gene in Maize
Source: Front Genet. 2016 Jul 26;7:131. doi: 10.3389/fgene.2016.00131 (PMC4960258; doi:10.3389/fgene.2016.00131)
Supplement: FIGURE S1 — ZmVDE1 gene structure based on the genomic sequence from maize inbred line B73. The red, sky blue, and yellow represent the transcription start and termination position, presumptive motif and coding sequence of ZmVDE1. [file Data_Sheet_1.DOC]

Resequence start

1 ACCCACCAAG CAGAGACGGA AAAGGAGGAT CGCTGGCTAA CCTATGAATG GCGGACGCAT

61 CGAGGGGCGC CTCGTCGGGA CCTTGCGCCG GTACTAGGGC TTGCGCCTCC GCCATGGCCG

121 TCTCACTCTC CCGCCTAGTA GACAGAGAGA TTTGGTTTTC GAAACGGAGG GGAGAAGGAA

G-box

181 AGCGAGCACG TGACTAACGT GAGACAGATG AACGACTGCG ATTCGTCCGG GCATGCCCGA

241 CTCCCTTCAG CCGGATGCCT GGACTGGATC CGTTTGCGCT TAACGGGGCC CCGTTTGGTC

I-box 5’UTR

301 GGCCCGTGAT AAGCAGGAGG CCAGGAGCAG CCGCCAGCCA GCTAGCAGCG TCCCTGCTGC

361 TCCGCGCCAC ACCTCTCCGC TTCATTCCTA TCCGTCTCCC CTGCTCTATC GTCGTCCTCC

421 TCCGTCGGGC CGGAGGAGCG TCTCTTTGTC GGCGCAGCGT GCAGAGCACC GCAGGTTCGG

481 CTCTTTGGCT TGCTCATCCC TTCCCCTCCT CTCCTAGGTT CTCCATCCCG CGCCACCTTC

541 TTGTTCCAGT TGCGTCTGCA TTCAGGGCTA CGTAGCATCA TTAGACGAAG GTTTCGTGCG

GT1-Motif

601 CGGTTAATCA TGCTTGGAAG TGAGAATTTT GTGACCCGGA TCGATCTTGG CGCATATTTT

GATA-Motif

661 GGACCTAGTG ATTGCGAGGA TATAAACGCG CTAGAATTCA GCTGCGGACA CTTCTTGTCA

721 GATGTCTGGC GGCAGATTGG TAGTTCAGTC TTATTAATAA AATAAAACAA AAAAGAGATG

781 AGTCATGGCT GCAATCCATA AACACAAACT GAGAATGTAC TCTAATGTTT ATCTAGAGAA

841 AGCCTGAAAC TTTTTCTGCG AACATTTCTG CTTGAATTGG TTTGAGTTCT ATACTGAACT

Translation Start

901 TTCCTCATGA GCTGCAGATT CAGAGACACG ATGATGTCGC GACAGTGTGC AAACCGCATC

1st exon

961 TTTCTTACCG GAGGTTCCAG CTTCCTCCAT GGCCTCAAAT CTAGAGTTGC ATCCAGTAGG

1021 AGTCACGGCA CCGTCAGACT CAACTGGCGC TGCGTGAGAA CTAATCTCTG GAGGGCCGAG

1081 CGCCTTGGTG TCAAGGCTAC TCCGTCTGAG GTAATACGAT TGCTTATTCG TTGCTACTGA

1141 CCAATTCACC TCTTGTTATA CCACTAAGAA GTAAGAACAG CAAGTTTTTG TCTCCTCCAG

2nd exon

1201 CATATCATTG CAGTACTGCA AGCGTCAGAT ATTTTCAGTG GCATCAGGAA ATGGAGCAAG

1261 TTGCAATTGG TCGCCATGAC AGGGGTGATG GCATGTGTAG TTCTGGTAGT CCCTTCTTCT

1321 GATGCAGTCG ATGCTCTCAA GACATGCACT TGCCTGCTAA AGGAATGCAG GTGACTCTTC

1381 ATCATACTGT CAACATAGTT TTGACACCAT ATAGGGGCTG CTTGNNNNNN NNNNNNNNNN

1441 NNNNNNNNNN NNNNNNNNNN NNNNNNNNNN NNNNNNNNNN NNNNNNNNNN NNNNNNNNNN

1501 NNNNNNNNNN NNNNNNNNNN NNNNNNNNNN NNNNNNNNNN TTTATATTCA GTATGTAGCC

1561 AATATAGGTC AAGAAGAAGT TAGTTACTAC TTGAAACTAA GAGCTCCCCG ATGACAAGTT

TCT-Motif

1621 TGTCATCTTT TGGATGAGAG TAACCTTAGA TCATTTTATA CATTTCTTAC TCAATTAGTT

1681 TCTCTCTCTC TCTTTCAGAA TAGAGCTGGC TAAATGCATA GCGAACCCAT CCTGTGCAGC

3rd exon

1741 AAACGTAGCA TGCTTGAACA CGTGCAACAA TCGACCTGAT GAAACTGAAT GTCAGGTTAT

1801 TCCCATAAAC CTTGAGCTCA CTGAAATCTG CCAGAAAAAA TAAGTTTTTT ATGCCCTGCT

4th exon

1861 TTTGTCATCC ATCAGATAAA ATGCGGAGAC CTGTTTGAGA ACAGTGTAGT CGATGAATTC

1921 AATGATTGTG CTGTTTCACG CAAGAAATGT GTTCCAAAAA AATCCGACGT CGGCGAGTTC

1981 CCCGTCCCCG ATCCATCTTC GCTGGTTAAG AGCTTCAACA TGGCAGATTT TAATGGCAAA

2041 TGGTACATTT CAAGTGGCTT AAACCCGACG TTTGACACAT TCGATTGTCA GCTTCACGAG

2101 TTTCATGTCG AGGGAGACAA ATTGATCGCG AACATCACAT GGAGAGTCCG CACCCCAGAC

2161 TCTGGCTTCT TTACCAGGTC AACCGTGCAG CGTTTTGTGC AGGACCCATC GCAGCCCGGC

2221 ATACTCTACA ACCATGACAA TGAGTTCCTG CACTATCAAG ATGACTGGTA AACCCTCTTA

MNF1-Motif

2281 GAAATTCTTT CTCACTACTG CCAATGTGCC CATTCCTCAA GGTTTATCGA CTTGGTATTT

2341 GCTGACCTGT AGGTACATTA TCTCATCCAA GGTTGAGAAC AAGGATGACG ACTACATATT

5th exon

2401 TGTGTACTAT CGTGGGCGAA ATGACGCATG GGATGGTTAT GGTGGTTCTG TATTGTACAC

2461 AAGAAGTAAA ACTGTACCTG AAACAATAAT ACCGGAGCTG GAAAGAGCTG CAAAGAGCGT

2521 AGGTCGGGAC TTCTCGACGT TCATCATGAC CGACAACACC TGTGGTCCTG AGCCTCCTCT

2581 TGTGGAGAGA ATCGAGAAAA CTGTGGTGGA AGGAGAGAAG ACCATCGTCA GGGAGGTGAA

2641 GGAGATCGAG GAGGAGGTTG AGGAGCTGGA GAAGGAGGAG GCGTCACTGT TTCAGAAGCT

2701 GGCAGAAGGT CTCATGGAGG TGAAACAGGA TTTCATGAAC TTCTTGCAGG GGCTGAGCAA

2761 GGAGGAGATG GAGCTGTTGG ATCAGCTCAA CATGGAAGCG ACTGAAGTTG AGCAAGTCTT

Stop codon

2821 CAGCCGTGCA CTACCGTTGA GGAAGCTAAG GTAGCTGCAG AGCAAAGGAC TTGGAGAAAA

3’UTR

2881 CAAGCCATCA AGTACTGGCC GAAACGGATT CTATTTGCCA CAGCAGTACC CACAGTAGAG

2941 ATTGTTGAAC AGATACAACA AACATTTTTA ATTTT--CTT TTCTTTTTCT TTTCTTTTCT

3001 TTTCTTTTCT TTGTTTACCC TTTTCTGTGG TCAAAACACA TGCCTACGTT AGCCCTATTT

3061 GTAAAGCATC AAGAGACAAC GTAACTTTTT CTTGCCAAAC AGAGAAAAAA AACAAGTGTA

3121 CTTAAGACTA CGCTGACCAT CTCTTGCATG ACAATTGTTT GTTGTTATAG TTTCCTTTGC

3181 CATTCCATCT AAATTTAAGT CATTTCCCTA TGTAAAATGC AAAATCAAAA GGAACTCTTA

3241 TATTTGTACG CCGTAGTGCA GTAGGGGACC TCTACCCCAG TTTTAAAGTA GAAGTAAAAA

3301 ACGGTATAGT TACATATCAC CGAATTTCAT GGGGTTTTTT AAGGAGACTA TGGAAAAGTT

3361 GATTATACTA AAAATCTTCT TCAGGAAGAC ACCAACTAGA AAAATGCTAG TAAAAGGATA

3421 ACTGGTTAAC TTGTATGGAT TTTACTAGCT GTGGTATGCA GTATTTTTTG TGTTTATAAT

3481 CTTATCATAG CAATTAAAAA TAACAAGGAA GCACACTGTA GCTTATTTCT AAATACTCCT

3541 TTTGTCCCAA ACTAAAATTT ATTTTAGATA ATTAATAGTA CATTTATACA ATATTGGATT

3601 TATGTATTTT ATATATGTGT CTAAATTCCA TTTGAATATA GATAAAAAAA CGAGCTAAAA

3661 CGAATGCTAT TTTGGGATGG AGGGAGTATA TTTTATTTTC GAACGCCCCA GTTGGTATGC

Resequence stop

3721 ATGTCA

**FIGURE S1  *ZmVDE1* gene structure based on the genomic sequence from maize inbred line B73.** The red, sky blue and yellow represent the transcription start and termination position, presumptive motif and coding sequence of *ZmVDE1*.
