# Supplementary figures and images for: Genetic Diversity and Molecular Evolution of a Violaxanthin De-epoxidase Gene in Maize
Source: Front Genet. 2016 Jul 26;7:131. doi: 10.3389/fgene.2016.00131 (PMC4960258; doi:10.3389/fgene.2016.00131)

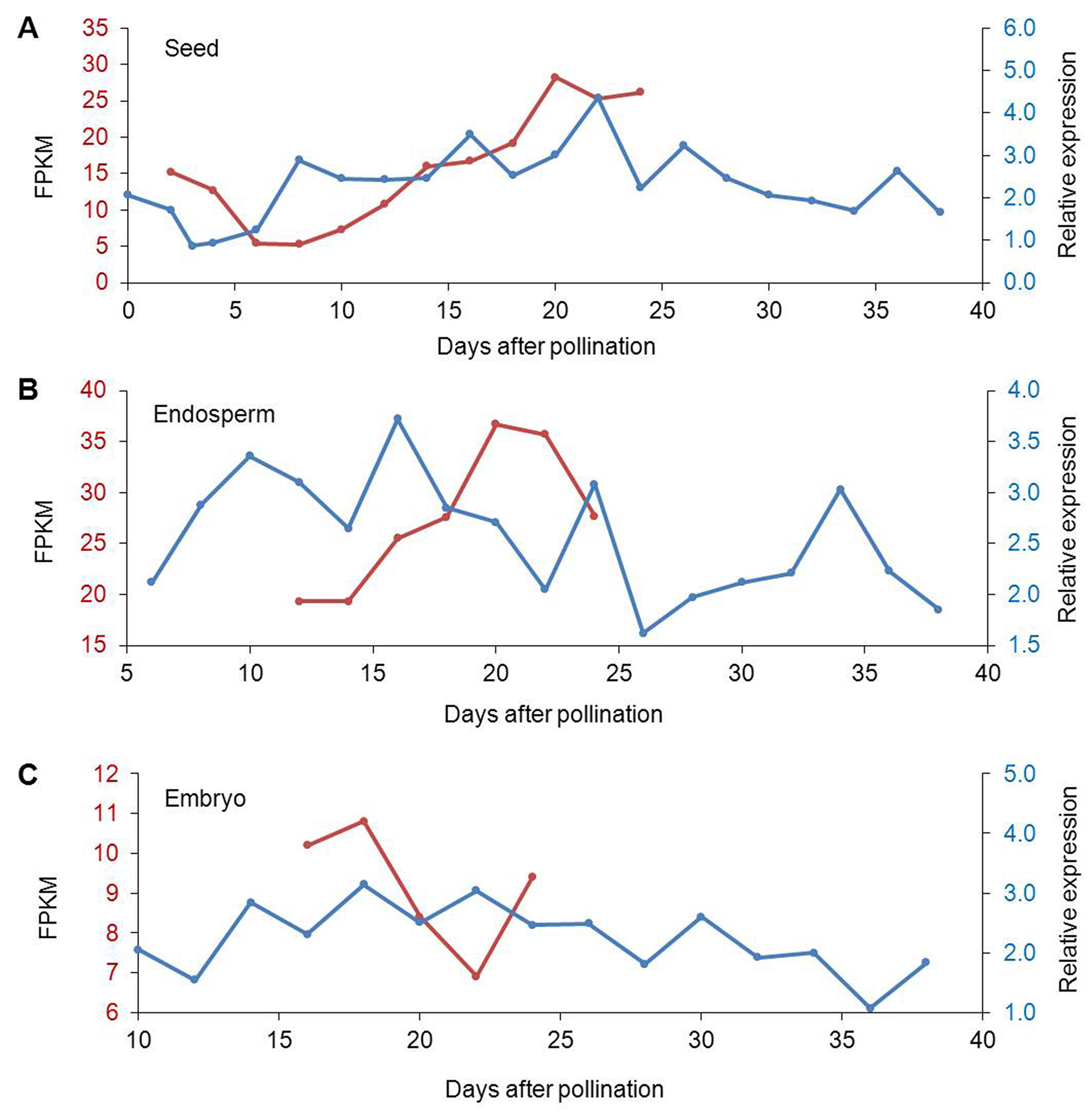

Supplement: FIGURE S2 — Expression patterns of ZmVDE1 in various tissues at different developmental stages in maize line B73. FPKM (red; Stelpflug et al., 2015) and relative expression (blue; Chen et al., 2014) represent the expression level of ZmVDE1 based on these two methods. (A–C) Expression of ZmVDE1 in seed (A), endosperm (B) and embryo (C). FPKM, fragments per kilobase of transcript per million mapped reads. [file Image_1.JPEG]

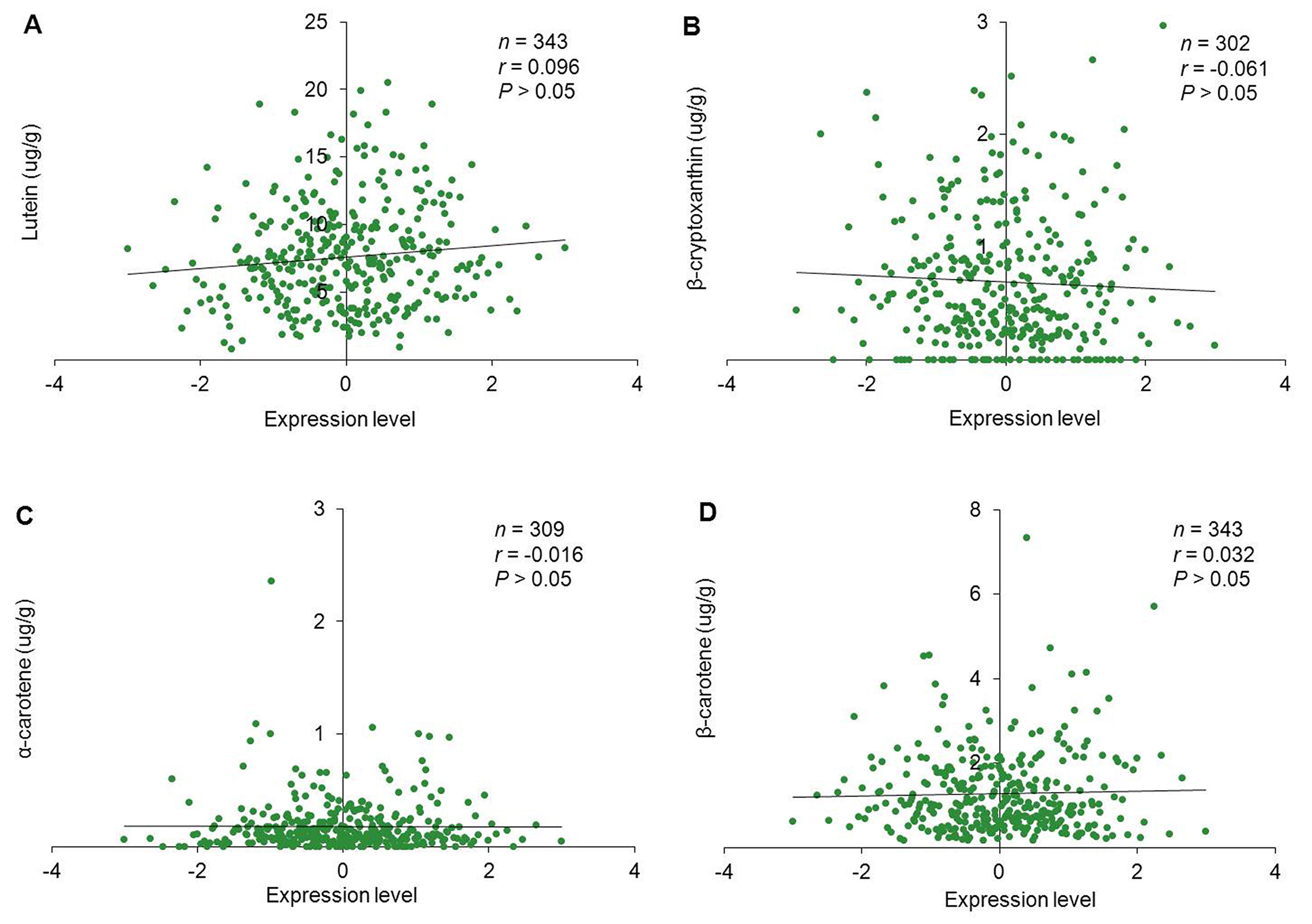

Supplement: FIGURE S3 — Plots of correlation between ZmVDE1 expression and carotenoid-related traits in maize kernels. (A–D) The x axis represents the normalized expression of ZmVDE1 in kernels collected at 15 DAP. The y axis represents the level of lutein (A), β-cryptoxanthin (B), α-carotene (C) and β-carotene (D). The r value is a Pearson correlation coefficient. [file Image_2.JPEG]

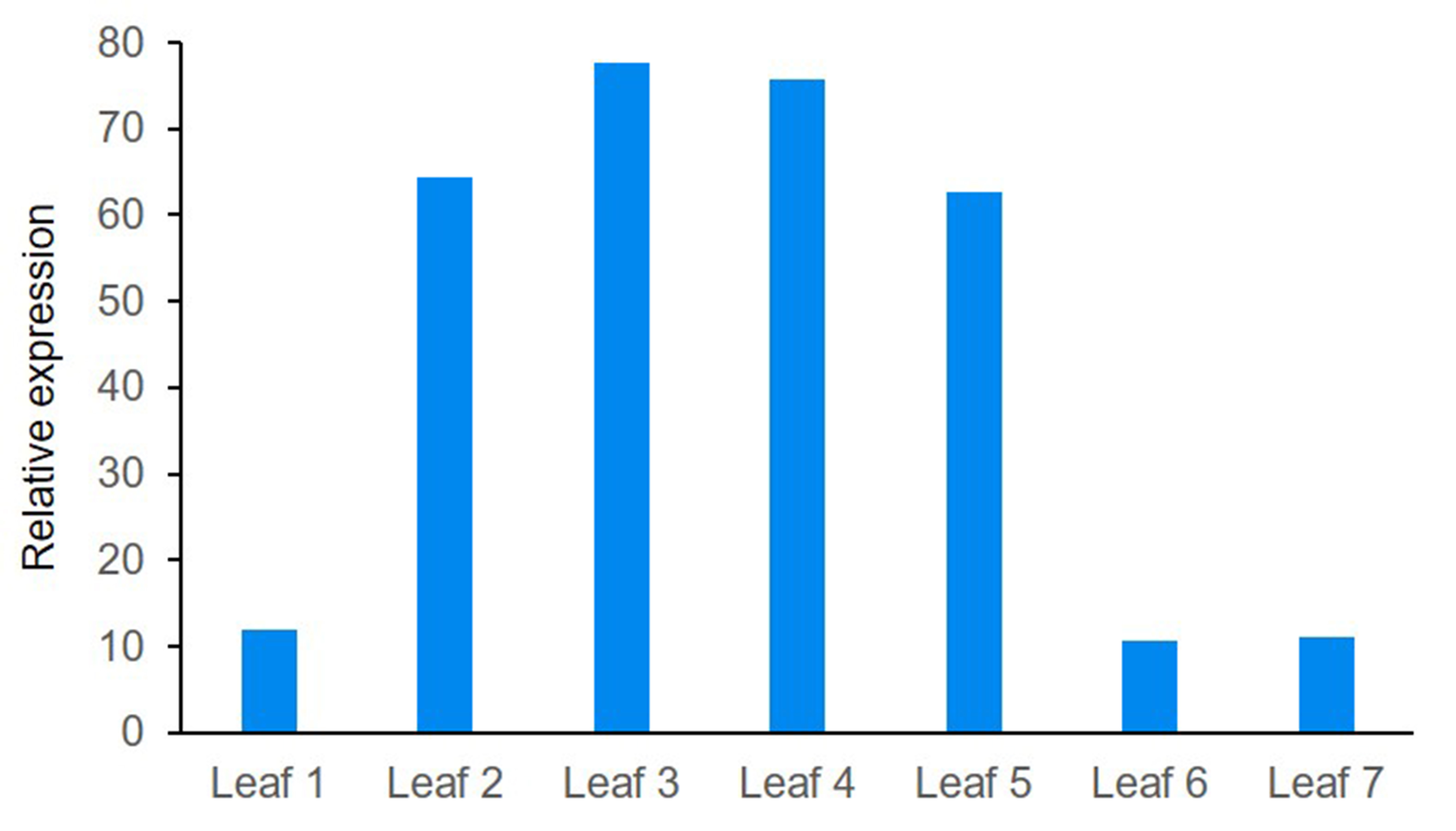

Supplement: FIGURE S4 — Expression patterns of ZmVDE1 in maize leaf in maize line B73. Histogram represent the expression level of ZmVDE1 based on Chen et al. (2014). [file Image_3.JPEG]
